# Characterization and phylogenetic analysis of the complete plastome of *Alopecurus japonicus* (Gramineae), an annual weed

**DOI:** 10.1080/23802359.2019.1704189

**Published:** 2020-01-07

**Authors:** Xiu-Xiu Guo, Chen Dai, Rong Wang, Xiao-Jian Qu, Xue-Jie Zhang

**Affiliations:** Key Lab of Plant Stress Research, College of Life Sciences, Shandong Normal University, Ji’nan, PR China

**Keywords:** *Alopecurus japonicus*, plastome, phylogenomics

## Abstract

*Alopecurus japonicus* is a weed in summer crop field, which is harmful to wheat crops. The complete plastome of *A. japonicus* was reported in this study. The genome was 136,408 bp in length, consisting of an 80,512 bp large single-copy region, a 12,836 bp small single-copy region, and two 21,530 bp inverted repeat regions. The GC content of this plastome was 38.3%. A total of 112 genes were annotated for the plastome of *A. japonicus*, containing 78 protein-coding genes (PCGs), 30 tRNAs, and 4 rRNAs. Phylogenetic analysis showed that *A. japonicus* was sister to *Alopecurus aequalis*.

*Alopecurus japonicus* is a one-year herb distributed in China, Japan and the Democratic People’s Republic of Korea, mainly in fields and wetlands at low altitudes. It belongs to the Gramineae family as rice, wheat, maize, and sweet sorghum (Bai et al. 2016; Deng et al. 2016; He et al. 2016; Ding et al. 2018; Li et al. 2018). *Alopecurus japonicus* is a weed in the summer crop field, which is harmful to wheat crops. At present, many studies are focused on its resistance to pesticides (Yang et al. 2007; Mohamed et al. 2012; Wu et al. 2016; Chen et al. 2018). Phylogenetically, *A. japonicus* belongs to genus *Alopecurus*, and there is still a big controversy about the systematic position of *Alopecurus.* Some studies advocated that *Alopecurus* should be placed in *Aveneae* or *Agrostideae* (Hitchcock and Chase 1935; Watson et al. 1986; Hilu and Esen 1990). Tzvelev suggested placing *Alopecurus* in *Phleae* (Tzvelev 1989). In this study, we showed the plastome of *A. japonicus*, which would provide a fundamental genetic resource for studying this important species.

Fresh leaves of *A. japonicus* were collected from Wanghui Village (Shandong, China; 36°31′N, 115°58′E). Voucher specimen (No.75) has been deposited at College of Life Sciences, Shandong Normal University. Modified CTAB method was used for plant total DNA extraction (Wang et al. 2013). Library preparation and sequencing were performed on the Illumina MiSeq platform at Novogene (Beijing, China). Organelle Genome Assembler (OGA, https://github.com/quxiaojian/OGA) was used to do plastome assembling (Qu 2019). Annotation was performed by using Plastid Genome Annotator (PGA, https://github.com/quxiaojian/PGA) (Qu et al. 2019). Geneious version 9.1.4 was used for manual annotation correction (Matthew et al. 2012). In order to determine the phylogenetic position of *A. japonicus*, a maximum-likelihood (ML) tree was reconstructed by RAxML version 8.2.10 (Alexandros 2014), using the alignment matrix of 78 protein-coding genes (PCGs) generated by MAFFT version 7.313 (Kazutaka and Standley 2013), the 1000 rapid bootstrap replicates, and the GTRGAMMA substitution model.

The complete plastome of *A. japonicus* (GenBank accession number: MN422307) was 136,408 bp in length, consisting of a large single-copy region (80,512 bp), a small single-copy region (12,836 bp), and a pair of inverted repeats regions (21,530 bp). The GC content of this plastome was 38.3%. 112 unique genes were encoded, including 78 PCGs, 30 tRNAs, and 4 rRNAs. The ML phylogenetic tree showed that *A. japonicus* was closely related to *A. aequalis* (Figure 1).

**Figure 1.**
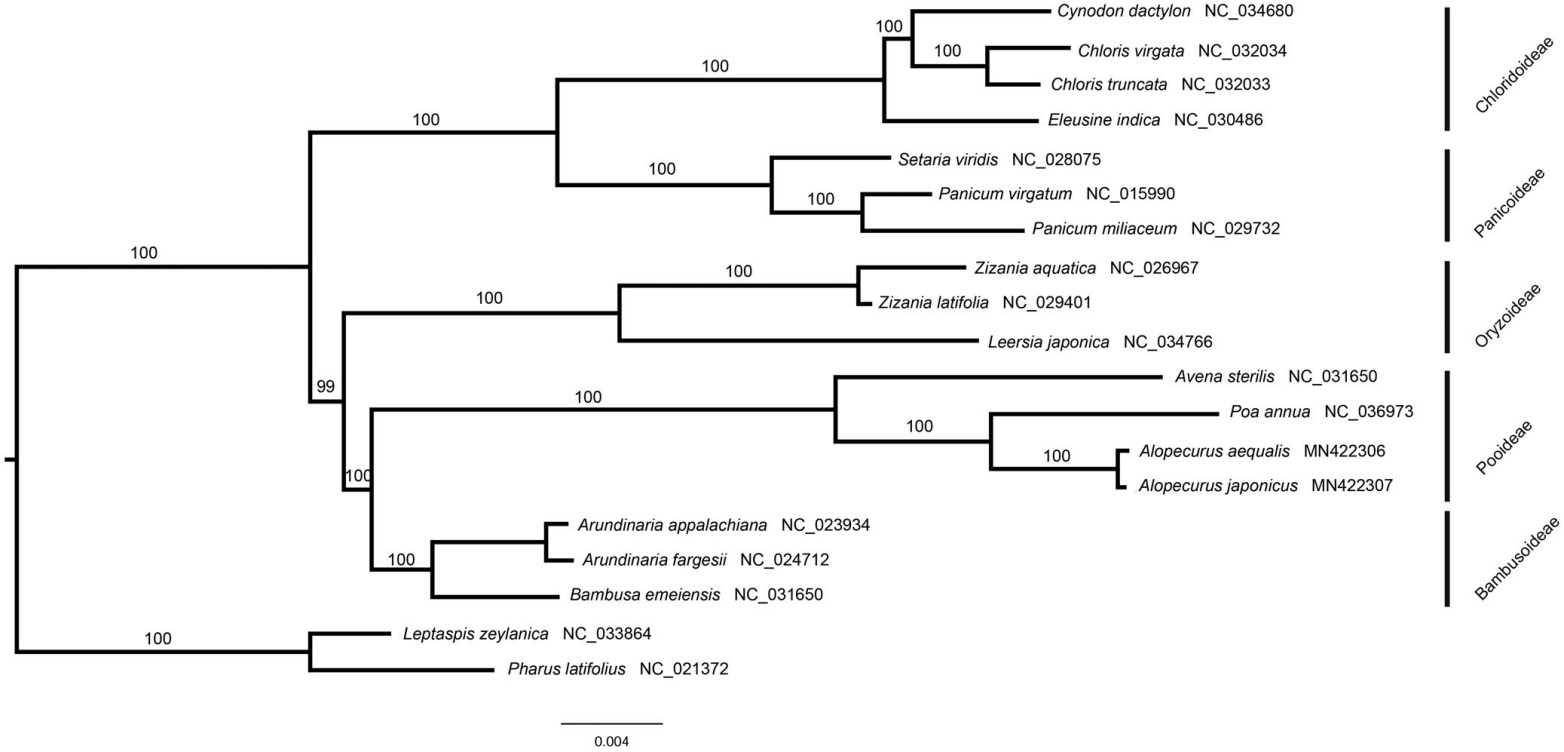
The maximum likelihood (ML) tree was reconstructed by 78 plastome genes. *Leptaspis zeylanica* and *Pharus latifolius* are used as out-group. Bootstrap support values are indicated on the branches of the ML tree.
